# The Epidermal Growth Factor Receptor (EGFR) Inhibitor Gefitinib Reduces but Does Not Prevent Tumorigenesis in Chemical and Hormonal Induced Hepatocarcinogenesis Rat Models

**DOI:** 10.3390/ijms17101618

**Published:** 2016-09-23

**Authors:** Silvia Ribback, Verena Sailer, Enrico Böhning, Julia Günther, Jaqueline Merz, Frauke Steinmüller, Kirsten Utpatel, Antonio Cigliano, Kristin Peters, Maria G. Pilo, Matthias Evert, Diego F. Calvisi, Frank Dombrowski

**Affiliations:** 1 Institut für Pathologie, Universitätsmedizin Greifswald, 17489 Greifswald, Germany; verena.sailer@ukb.uni-bonn.de (V.S.); e.boehning@yahoo.de (E.B.); guenther.julia@hotmail.de (J.G.); jacqueline.merz@gmx.de (J.M.); f.steinmueller@diako-online.de (F.S.); kirsten.utpatel@klinik.uni-regensburg.de (K.U.); a.cyglius@gmail.com (A.C.); tp905@web.de (K.P.); giuliapilo1983@gmail.com (M.G.P.); matthias.evert@uni-greifswald.de (M.E.); dcalvisi@uni-greifswald.de (D.F.C.); frank.dombrowski@uni-geifswald.de (F.D.); 2 Englander Institut for Precision Medicine, Weill Cornell University of Medicine, New York, NY 10065, USA; 3 Pathologisches Institut Diakonie-Krankenhaus, 27356 Rotenburg (Wümme), Germany; 4 Institut für Pathologie, Universitätsklinikum Regensburg, 93053 Regensburg, Germany

**Keywords:** hepatocarcinogenesis, intraportal transplantation, epidermal growth factor receptor (EGFR), Gefitinib

## Abstract

Activation of the epidermal growth factor receptor (EGFR) signaling pathway promotes the development of hepatocellular adenoma (HCA) and carcinoma (HCC). The selective EGFR inhibitor Gefitinib was found to prevent hepatocarcinogenesis in rat cirrhotic livers. Thus, Gefitinib might reduce progression of pre-neoplastic liver lesions to HCC. In short- and long-term experiments, administration of *N*-Nitrosomorpholine (NNM) or intrahepatic transplantation of pancreatic islets in diabetic (PTx), thyroid follicles in thyroidectomized (TTx) and ovarian fragments in ovariectomized (OTx) rats was conducted for the induction of foci of altered hepatocytes (FAH). Gefitinib was administered for two weeks (20 mg/kg) or three and nine months (10 mg/kg). In NNM-treated rats, Gefitinib administration decreased the amount of FAH when compared to controls. The amount of HCA and HCC was decreased, but development was not prevented. Upon all transplantation models, proliferative activity of FAH was lower after administration of Gefitinib in short-term experiments. Nevertheless, the burden of HCA and HCC was not changed in later stages. Thus, EGFR inhibition by Gefitinib diminishes chemical and hormonal also induced hepatocarcinogenesis in the initiation stage in the non-cirrhotic liver. However, progression to malignant hepatocellular tumors was not prevented, indicating only a limited relevance of the EGFR signaling cascade in later stages of hepatocarcinogenesis.

## 1. Introduction

Hepatocellular carcinoma (HCC) is the fifth leading cause of tumor related deaths worldwide with increasing incidence in the Western hemisphere [1]. Main causes are chronic hepatitis B and C virus infection, long-term alcohol abuse, aflatoxins in foodstuff, and rare inherited diseases such as alpha-1 antitrypsin deficiency and hemochromatosis [2].

Furthermore, hormonal influences like hyperinsulinism in type 2 diabetes mellitus and long-term intake of oral contraceptives are risk factors for hepatocarcinogenesis in the non-cirrhotic liver [3]. The prognosis and overall survival of HCC patients is poor, with less than 5% five-year survival. Partial resection and liver transplantation remain the only curative options, but most cases of HCC are diagnosed in an advanced stage and are not suitable for surgical therapy. Ablation therapies, including radio-frequency ablation, transarterial chemoembolization, and radioembolization are effective in slowing tumor progression in advanced stages [4]. HCC is extremely resistant to conventional chemotherapy [5]. New hopes come from the use of targeted therapies, such as receptor tyrosine kinase inhibitors. Sorafenib (Nexavar^©^, Bayer, Karlsruhe, Germany) was the first (and still the only one) of this class of new drugs to be approved by the Food and Drug Administration for the treatment of HCC, as overall survival was improved [6].

The epidermal growth factor receptor (EGFR) is a transmembraneous protein with intrinsic tyrosine kinase activity and belongs to the family of erbB proteins. Binding of EGF or transforming growth factor α (TGFα) leads to receptor dimerization and autophosphorylation of the intrinsic tyrosine kinase [7,8].

Downstream signaling cascades include the rat sarcoma/rat sarcoma-activated factor/mitogen activated protein kinase/extracellular regulated kinase kinase (Ras/Raf/MEK/ERK) and the phosphatidyl-inositide 3 kinase/protein kinase B/mammalian target of rapamycin (PI3K/AKT/mTOR) pathways [9]. Immunohistochemical analysis often reveals co-localization of EGFR and TGFα in human HCC, supporting the hypothesis of autocrine and paracrine mitogenic action of TGFα to hepatocytes [10].

Gefitinib (Iressa^®^, Astra-Zeneca, Wedel, Germany) is a small molecule designed to block the catalytic domain of the EGFR tyrosine kinase, thus inhibiting downstream signaling [11]. The results of a contemporary Phase II trial approaching the adjuvant Gefitinib administration after local ablation of HCC are still pending [12]. Applying these small molecules in models of hepatocarcinogenesis would lead to both a deeper understanding of the molecular pathogenesis of HCC and the evaluation of their efficacy for the treatment of this deadly disease. Continuous oral administration of *N*-Nitrosomorpholine (NNM) is a well-established model of chemically-induced hepatocarcinogenesis [13]. Earliest morphological changes in the liver are pre-neoplastic lesions (foci of altered hepatocytes (FAH)) that are of a clear cell or mixed cell appearance, which progress to hepatocellular adenoma (HCA) and carcinoma (HCC).

FAH can also be induced by hormonal stimuli—after intraportal transplantation of pancreatic islets in diabetic rats (PTx, [14]), ovarian fragments in ovarectomized rats (OTx, [15]) and thyroid follicles in thyroidectomized rats (TTx, [16]). After low-number islet transplantation, insulin related alterations lead to glycogen and fat storing clear cell foci downstream of the islet transplant, which evolve into basophilic foci and progress to HCA and HCC [14]. The hepatocytes downstream of the transplanted ovarian fragments are characterized by an amphophilic phenotype driven by estrogens [17], which also evolve to HCA and HCC [15]. Intraportal transplantation of thyroid follicles and the influence of triiodothyronine (T3) lead to hyperproliferative FAH downstream of the transplants, which are amphophilic-tigroid [16]. T3 is a direct mitogen for hepatocytes without preceding cell loss [18]. By contrast to the other models, a progression to HCA or HCC could not be seen in long term experiments after thyroid follicle transplantation.

Overexpression of TGFα is documented in NNM induced hepatocarcinogenesis [19] and pre-neoplastic lesions of PTx and OTx transplantation models [14,15], but not in T3 induced hepatocellular alterations [16].

Therefore, we investigated the antitumoral effect of Gefitinib in rat models of chemically-induced hepatocarcinogenesis (administration of NNM) and three different intraportal transplantation models of hormonal active tissue (PTx, OTx or TTx), with particular regard to early pre-neoplastic lesions (in the case of NNM, insulin and estrogens, respectively), and hyperproliferative hepatocellular alterations (in the case of triiodothyronine, T3).

## 2. Results

### 2.1. Hepatocellular Lesions after N-Nitrosomorpholine (NNM) Administration

**Figure 1:**
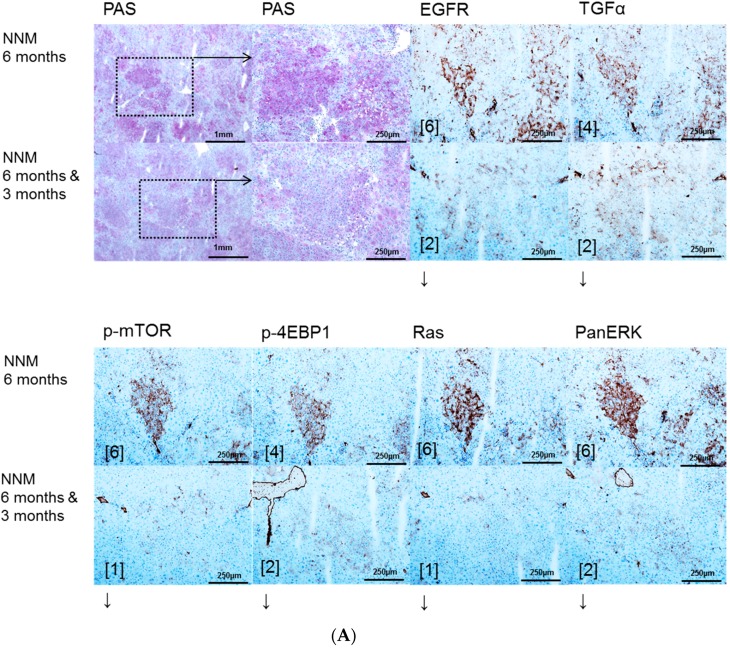

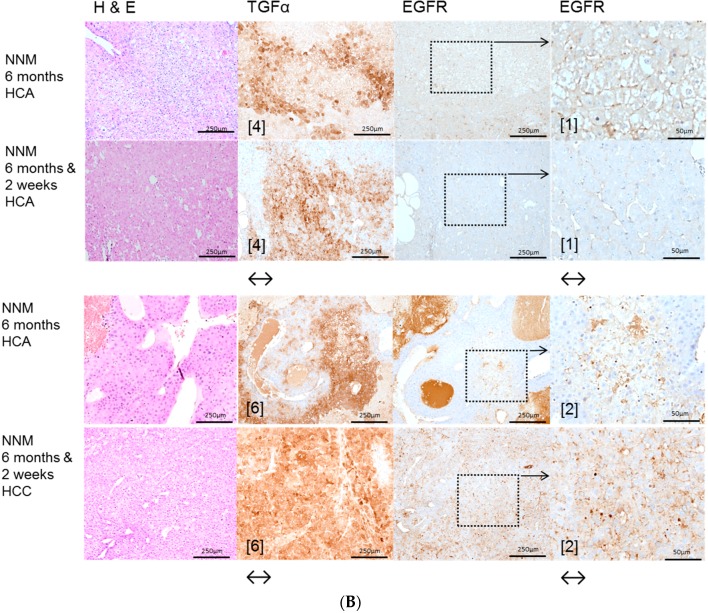
Effects of Gefitinib at six months after administration of *N*-Nitrosomorpholine (NNM) and expression patterns of epidermal growth factor receptor (EGFR), transforming growth factor α (TGFα) and downstream signaling in pre-neoplastic foci of altered hepatocytes (FAH) and neoplastic liver lesions. Gefitinib was administered daily for three months (10 mg/kg) (**A**); Glycogen storing foci of altered hepatocytes (FAH) after NNM administration (NNM 6 months) with an overexpression of EGFR and TGFα, which is reduced after Gefitinib treatment for three months (NNM 6 months & 3 months), implying downregulation of proteins of dependent signaling pathways of phosphatidyl-inositide 3 kinase/protein kinase B/mammalian target of rapamycin (PI3K/AKT/mTOR) (p-mTOR and phosphorylated 4E-binding protein 1 (p-4EBP1)) and rat sarcoma/rat sarcoma-activated factor/mitogen activated protein kinase/extracellular regulated kinase kinase (Ras/Raf/MEK/ERK) (Ras and PanERK) pathways, corresponding to less proliferative activity and volume fraction of FAH in Gefitinib treated rats (**B**). After six months of NNM administration (NNM 6 months), hepatocellular adenomas (HCA) and carcinomas (HCC) reveal a strong of TGFα, to a lesser extent of EGFR. Gefitinib treatment for two weeks or three months (NNM (6 months & 2 weeks)/(6 months & 3 months)) does not alter protein expression levels. Immunostaining scores are shown in brackets. Arrows correspond to ↑ upregulation, ↓ downregulation or ↔ unchanged expression level. Expression levels of immunostaining are given in brackets. Magnification: (**A**) overview 40×; marked lesion in dashed lines (PAS and immunostainings) 100×; and (**B**) (H & E and immunostainings) 100×; marked area in dashed lines (EGFR) 400×.

FAH were induced in all animals that received NNM for three months, and mainly consisted of clear cell foci, whereas HCA or HCC were not observed at this time point. After six months of NNM administration, multiple HCA and HCC were detected in all NNM-treated rats (Figure 1).

#### 2.1.1. Proliferative Activity

After three months of NNM administration, FAH of all NNM groups exhibited a three-fold higher BrdU-LI than the extrafocal liver tissue. Gefitinib treatment did not affect the proliferation rate in rats. After six months of NNM administration and Gefitinib therapy for three months, proliferative activity of FAH was slightly reduced compared to animals without Gefitinib treatment (BrdU-LI of NNM six months alone 85.50% ± 1.90%; NNM six months, Gefitinib three months 70.36% ± 1.75%; *p* < 0.05). BrdU-LI was not altered in HCA or HCC (Table 1).

#### 2.1.2. Volume Fraction of FAH and HCA

After NNM administration for three months, FAH foci occupied a reduced volume portion of liver parenchyma after two weeks Gefitinib with 4.92 ± 0.87 vol % and after three months with 1.58 ± 0.28 vol % as compared to administration of NNM alone (7.25 ± 0.90 vol %; *p* < 0.05).

After six months, volume fraction of FAH was not reduced after Gefitinib treatment. A halving of volume portion of HCA from 10.36 ± 0.89 vol % to 5.43 ± 0.57 vol % could be instead appreciated (*p* < 0.05). The number of HCC was reduced from 7.93 ± 0.92 to 4.87 ± 0.77 tumors per animal (*p* < 0.05) (Table 1).

### 2.2. Hepatocellular Lesions in Transplantation Models

#### 2.2.1. Short Term Experiments

**Figure 2:**
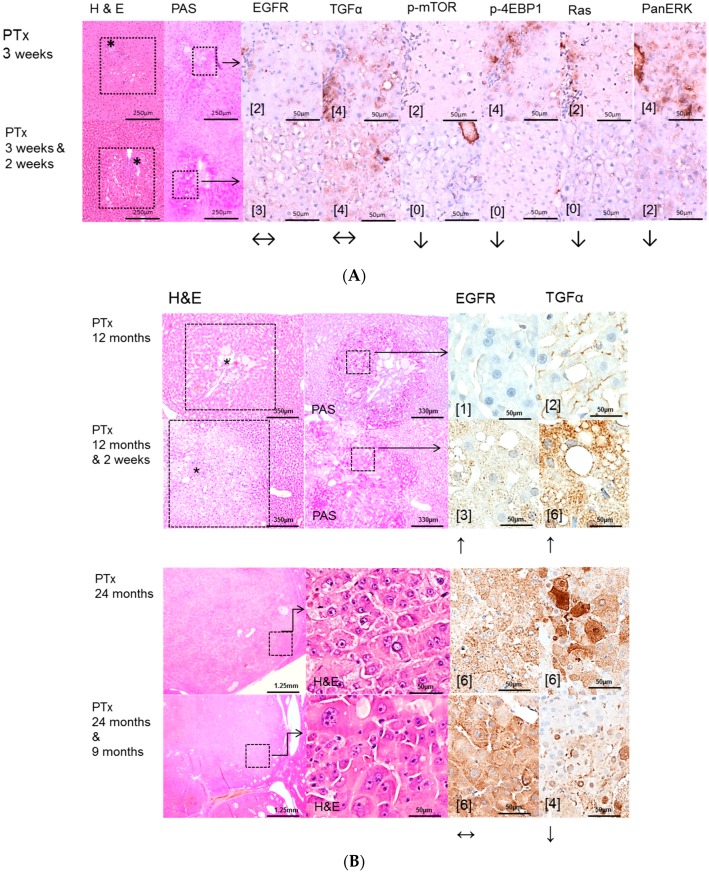

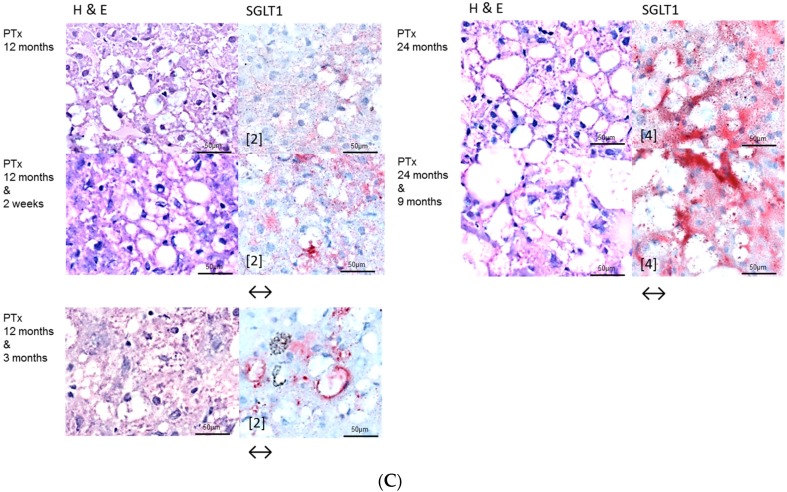
Effects of Gefitinib after intraportal transplantation of pancreatic islets in diabetic rats (PTx) and expression patterns of EGFR, TGFα, downstream signaling pathways and the glucose transporter protein Sodium-glucose cotransporter 1 (SGLT1) in pre-neoplastic foci of altered hepatocytes (FAH) and neoplastic liver lesions. Gefitinib was administered daily for two weeks (20 mg/kg body weight) or nine months (10 mg/kg), respectively. (**A**) **Short-term experiments** (three weeks and six months). FAH (dashed lines) of untreated rats (PTx 3 weeks and 6 months) at the downstream level of transplanted islets (*) with glycogen storage (PAS) with a slight overexpression of EGFR and TGFα. After Gefitinib treatment for two weeks (PTx 3 weeks & 2 weeks), only TGFα is upregulated. Nevertheless, downstream signaling with representative p-mTOR, p-4EBP1, Ras, and PanERK is downregulated corresponding to less proliferative activity after Gefitinib administration. By contrast, at six months (PTx (6 months & 2 weeks)/(6 months & 3 months)), signaling is upregulated or not altered respectively, suggesting resistance to Gefitinib; (**B**) **Long-term experiments** (12 and 24 months). After 12 months, expression levels of EGFR or TGFα are higher in FAH (dashed lines, with islets (*)) after Gefitinib treatment in comparison to untreated rats. After 24 months, hepatocellular carcinomas with overexpression of EGFR and TGFα, which is almost not reduced after Gefitinib treatment; (**C**) The SGLT1 glucose transporter is expressed in FAH of untreated PTx rats. There is no difference after Gefitinib treatment. Immunostaining scores are shown in brackets. Arrows correspond to ↑ upregulation, ↓ downregulation or ↔ unchanged expression level. Expression levels of immunostaining are given in brackets. Magnification: (**A**) 3 weeks/(3 weeks & 2 weeks) (H & E and PAS) 40×; marked area in dashed lines (immunostainings) 400×; 6 months/(6 months & 2 weeks)/(6 months & 3 months) (H & E, PAS) 200×; marked areal in dashed line (immunostainings) 400×; (**B**) 12 months/(12 months & 2 weeks) (H & E and PAS) 60×; marked area in dashed line (immunostainings) 400×; 24 months/(24 months & 9 months) overview 20×; marked area in dashed lines (H & E and immunostainings) 400×; (**C**) (H & E and SGLT1) 400×.

**Figure 3:**
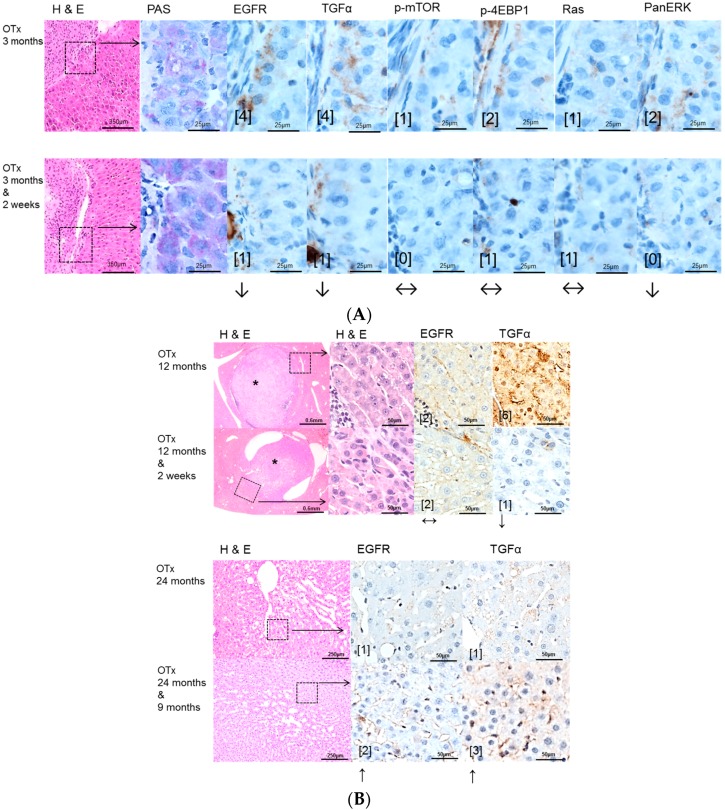
Effects of Gefitinib after intraportal transplantation of ovarian fragments in ovariectomized rats (OTX) and expression patterns of EGFR, TGFα and downstream signaling pathways in pre-neoplastic foci of altered hepatocytes (FAH) and neoplastic liver lesions. Gefitinib was administered daily for two weeks (20 mg/kg body weight) or 9 months (10 mg/kg), respectively. (**A**) **Short-term experiments** (three months). FAH of untreated rats (OTx 3 months) at the downstream level of transplanted ovarian fragments reveal loss glycogen storage (PAS) and a slight overexpression of EGFR and TGFα and also of downstream signaling with representative p-mTOR and PanERK, p-4EBP1 and Ras. After Gefitinib treatment for two weeks (OTx 3 months & 2 weeks), the EGFR and TGFα but not downstream signaling are slightly reduced; (**B**) **Long-term experiments** (12 and 24 months). Transplant tumors and associated FAH with unaltered EGFR but downregulated TGFα expression level after Gefitinib treatment for two weeks (OTx 12 months & 2 weeks) in comparison to untreated rats (OTx 12 months). Small HCAs (*) with upregulation of the EGFR and TGFα after Gefitinib treatment for 9 months (OTx 24 months & 9 months). Immunostaining scores are shown in brackets. Arrows correspond to ↑ upregulation, ↓ downregulation or ↔ unchanged expression level. Expression levels of immunostaining are given in brackets. Magnification: (**A**) 3 months/(3 months & 2 weeks) (H & E) 40×; marked area in dashed lines (PAS and immunostainings) 600×; (**B**) 12 months/(12 months & 2 weeks) (H & E) 40×; marked area in dashed line (H & E and immunostainings) 400×; 24 months/(24 months & 9 months) (H & E) 100×; marked area in dashed lines (immunostainings) 400×.

**Figure 4:**
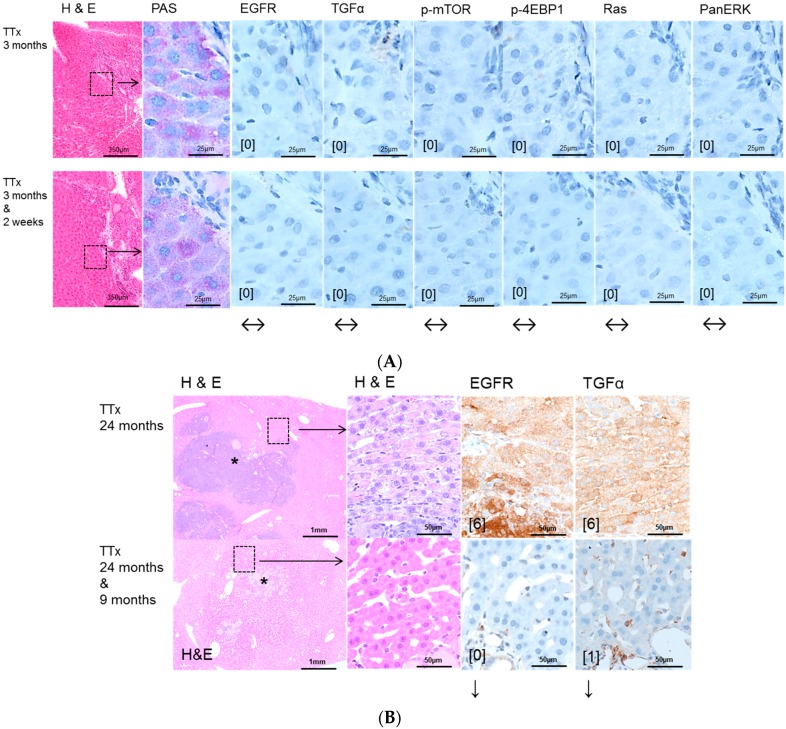
Effects of Gefitinib at three months after intraportal transplantation of thyroid follicles fragments in thyroidectomized rats (TTx) and expression patterns of EGFR and TGFα and downstream signaling pathways in pre-neoplastic foci of altered hepatocytes (FAH) and neoplastic liver lesions. Gefitinib was administered daily for nine months (10 mg/kg). (**A**) **Short-term experiments**. FAH of untreated rats (TTx 3 months) at the downstream level of transplanted thyroid follicles reveal loss glycogen storage (PAS). There is no overexpression of EGFR and TGFα or of downstream signaling with representative p-mTOR, p-4EBP1, Ras and PanERK. After Gefitinib treatment for two weeks (TTx 3 months & 2 weeks), the EGFR and TGFα expression and downstream signaling is also not upregulated and thus not altered; (**B**) **Long-term experiments** (24 months). Transplanted follicles (*) and associated hepatocellular adenoma with strong overexpression of EGFR and TGFα of untreated rat (TTx 24 months) in comparison to treated rat (TTx 24 months & 9 months) with downregulation of EGFR and TGFα. Immunostaining scores are shown in brackets. Arrows correspond to ↑ upregulation, ↓ downregulation or ↔ unchanged expression level. Expression levels of immunostaining are given in brackets. Magnification: (**A**) 3 months/(3 months & 2 weeks) (H & E) 40×; marked area in dashed lines (PAS and immunostainings) 600×; (**B**) 24 months/(24 months & 9 months) (H & E) 40×; marked area in dashed lines (H & E and immunostainings) 400×.

Intraportal transplantation of pancreatic islets, ovarian fragments, and thyroid follicles resulted in the development of FAH in the transplanted rats (Figure 2A, Figure 3A, Figure 4A). In particular, typical clear cell foci—demonstrated by the PAS reaction—developed after islet transplantation, whereas amphophilic, glycogen-depleted foci occurred after transplantation of ovarian fragments, and amphophilic-tigroid cell foci followed the transplantation of thyroid follicles. FAH exhibited increased proliferative activity in comparison to unaltered extrafocal liver tissue. The proliferative activity of FAH was reduced upon all transplantation models after administration of high-dose Gefitinib (20 mg/kg body weight). Indeed, the BrdU-LI was approximately halved in the Gefitinib-treated groups (Gefitinib treatment vs. untreated: PTx six months 2.27% ± 0.13% vs. 6.06% ± 0.49%, *p* < 0.05; OTx three months: 12.67% ± 2.67% vs. 29.79% ± 2.68%, *p* < 0.05; TTx three months: 7.69% ± 1.07% vs. 12.25% ± 1.33%, *p* < 0.05). Gefitinib administration had almost no effect on proliferation of unaltered hepatocytes in the extrafocal liver parenchyma (Table 2).

#### 2.2.2. Long Term Experiments

Administration of high dose Gefitinib for two weeks before sacrifice could significantly reduce the proliferative activity only in FAH from the OTx model after 12 months (8.73% ± 0.88% vs. 16.52% ± 2.47%, *p* < 0.05, Table 3). Low dose treatment with Gefitinib for three months or nine months before sacrifice did not influence the proliferation rate of FAH or extrafocal liver tissue in the three transplantation models in 12 and 24 months experiments. Frequency and quantity of FAH did not differ between treated and untreated groups. Transplant associated HCA arose in all three transplantation models after 12 and 24 months post-transplantation without Gefitinib as well as after high (two weeks prior to sacrifice) or low dose (three or nine months before sacrifice) Gefitinib treatment (Figure 2B, Figure 3B, Figure 4B). In general, 12 months after transplantation, HCA occurred most frequently in PTx livers. After 24 months, HCA could also be detected in OTx and TTx animals. Transplant associated HCC arose very seldom and only after 24 months after transplantation in PTx and OTx animals without and with high dose of Gefitinib. Hyperplasia of transplants developed often after OTx and transplant tumors in some cases after TTx, in untreated and Gefitinib-treated rats at both time points. Transplant tumors of TTx livers mainly revealed follicular or papillary morphology with mild to moderate atypia; in one case (24 months, without Gefitinib), criteria for a thyroid carcinoma with invasive growth and high mitotic activity were applicable (Table 3).

### 2.3. Effects of Gefitinib on Expression Levels of EGFR, TGFα and Downstream Effectors in Different Hepatocarcinogenesis Models

EGFR and TGF-α are upregulated in pre-neoplastic FAH, HCA and HCC induced after NNM (Figure 1) administration and after PTX (Figure 2) and OTx (Figure 3) in untreated rat livers in comparison to extrafocal liver tissue, whereas TTx (Figure 4) induced lesions revealed no overexpression. Representative downstream target proteins of the AKT/mTOR and MAPK signaling pathways (p-mTOR, p-4EBP1, Ras and PanERK) were also strongly activated, as expected, in NNM and PTx induced FAH, and also after OTx, but are not detectable after TTx.

Gefitinib treatment for three months reduced the expression levels of EGFR and TGFα in FAH of six months treated NNM rats, thus correlating with less proliferative activity and volume fraction of FAH in Gefitinib treated rats. Nevertheless, levels of AKT/mTOR and MAPK cascades were not altered in hepatocellular tumors (Figure 1).

While EGFR expression is not altered and TGFα is predominantly upregulated in the PTx model after administration of Gefitinib, the downstream AKT/mTOR and MAPK pathways were downregulated, corresponding to less proliferative activity in short time experiments. By contrast, at six months and Gefitinib treatment for two weeks/three months, downstream cascades were not altered or even upregulated, suggesting the resistance to Gefitinib. In long-term PTx experiments of 12 and 24 months, expression levels of EGFR or TGFα were also higher in FAH after Gefitinib treatment, as it has been detected in short-term experiments. HCCs showed strong overexpression of EGFR and TGFα, which was almost not reduced after Gefitinib treatment. Additional assessment of the transporter protein sodium/glucose cotransporter 1 (SGLT1), whose expression increases following EGFR inactivation [20] revealed cytoplasmatic and slight membranous expression in FAH of untreated PTx rats, which was not altered after Gefitinib treatment (Figure 2).

In early FAH, which are related to transplanted ovarian fragments of the OTX model, the EGFR and TGFα and also downstream signaling of MAPK and AKT/mTOR were found to be slightly upregulated, which could be only slightly diminished after Gefitinib treatment in three months and 12 months experiments. Thus, reduction of proliferative activity of FAH cannot be attributed to this signaling pathway inhibition, as epatocellular neoplasms, such as HCA after 24 months, also reveal upregulation of the EGFR and TGFα after Gefitinib treatment (Figure 3).

Early hepatocellular proliferative lesions of the TTx model did not express the EGFR, TGFα or downstream target proteins, neither without nor after Gefitinib administration (Figure 4). Upregulation in hepatocytes could only be seen in one animal at the downstream level in one animal with a malignant transplant tumor, and thus a single observation (not shown).

## 3. Discussion

Hepatocarcinogenesis is accompanied by overexpression of multiple growth factors and activation of various signaling pathways, including EGF, Vascular endothelial growth factor (VEGF), Insulin like growth factor (IGF), MAPK, PI3K/AKT/mTOR, and Wnt/β-Catenin [21,22,23,24,25,26]. They might represent valuable targets for systemic molecular-based therapies. EGFR is expressed in most HCC [10,27], and EGFR inhibitors such as the monoclonal antibody cetuximab or tyrosine kinase inhibitors such as Gefitinib and Erlotinib are able to suppress HCC growth both in vitro and in vivo [9,28,29].

In this study, we investigated the antiproliferative and antitumoral activity of the EGFR tyrosine kinase inhibitor Gefitinib in various models of hormonally- and chemically-induced hepatocarcinogenesis.

In the NNM model, representing chemical hepatocarcinogenesis without contemporary liver cirrhosis, selective EGFR inhibition by Gefitinib diminished hepatocarcinogenesis at the level of initiation (early time points) and progression (later time points), as the volume fraction of pre-neoplastic liver lesions and HCAs and the number of HCC were reduced. These results are supported by former findings of Gefitinib effects on sequential cirrhosis and HCC development in diethylnitrosamine treated rats [30], which was interpreted as a chemopreventive option for HCC development. Interestingly, EGFR inhibition by Gefitinib did not influence proliferative activity in NNM induced FAH, but the amount of pre-neoplastic and neoplastic tissue was reduced, accompanied by downregulation of the EGFR and TGFα expression levels and reduction of related protooncogenic signaling. Furthermore, the antitumoral effect of Gefitinib might be directed toward cells other than hepatocytes. In accordance with the latter hypothesis, it has been shown that Gefitinib administration leads to the inhibition of angiogenesis due to apoptosis induction in endothelial cells and decreased production of proangiogenic molecules in pancreatic cancer [31].

The protooncogenic signaling pathways of AKT/mTOR and Ras/raf-1/MAPK, which are activated in the NNM and the PTx [32,33,34], could also be substantiated to be slightly upregulated after OTx, but not after TTx. At the initiation level of FAH (short time experiments) inhibition of the EGFR by Gefitinib could reduce proliferative activity corresponding to a diminished downstream signaling in FAH after PTx and OTx.

On the contrary, Gefitinib administration did not influence proliferative activity of FAH or the development of hepatocellular neoplasms at the progression level in long-term experiments. Furthermore, downstream signaling pathways were upregulated after Gefitinib administration, suggesting resistance mechanisms in hepatocytes. One has to consider that, due to severe side effects, Gefitinib must be administered at a low dose in long-term experiments, which might partly explain the lack of inhibition of liver tumor development in the various models examined. Nevertheless, resistance to Gefitinib in the late stages of hepatocarcinogenesis is very likely and has been described in the treatment of human non-small-cell lung cancer as well [35] and in HCCs of diethylnitrosamine treated rats [30].

Possible resistance of the AKT/mTOR and Ras/raf-1/MAPK oncogenic signaling pathways to EGFR tyrosine kinase inhibitors has been described [30,36]. Activation of these pathways by other growth factor receptors like Hepatocyte growth factor/c-Met, IGF-1R and the Insulin receptor could explain the emergence of residual tumors [37,38,39,40,41]. Furthermore, other members of the erb-B receptor family, which are also upregulated in human HCC [22], might also drive hepatocarcinogenesis. The effect of Gefitinib to these receptors remains poorly understood.

Lately, Steinway [37] found that the EGFR pathway acts as a compensatory survival mechanism upon c-Met inhibition in human c-Met-positive HCC, and combined inhibition of both c-Met and EGFR oncogenic pathways provides superior suppression of HCC tumor growth.

We could substantiate an upregulation of the sodium/glucose cotransporter 1 (SGLT1) in FAH of PTX rats after Gefitinib treatment, another possible explanation of resistance with unrestrained proliferation and cell growth. SGLT1 maintains high intracellular glucose levels leading to cell survival and intracellular homeostasis, is activated by EGFR independent of its kinase activity, and is therefore not influenced by tyrosine kinase inhibitors [20].

Furthermore, our findings of upregulation of the EGFR expression levels, mainly in liver lesions after PTx, illustrate resistance to Gefitinib after long term application. Nevertheless, this paradoxical conversion of EGFR expression cannot be explained by direct effects of Gefitinib, as its target is only the catalytic domain of the EGFR tyrosine kinase [11] with inhibition of downstream signaling. Mechanisms of transactivation of the EGFR by other activated receptors in hepatocarcinogenesis, as has been shown for the Insulin-Like Growth Factor Receptor 1 (IGF-1R) in Erlotinib treated HCC cell lines Huh7 [42], would be a possible explanation.

In this study, some HCA could be detected after intraportal transplantation of thyroid follicles (TTx) for the first time in this experimental setting, as manifest hepatocellular neoplasms have not been described by Dombrowski et al. [16]. Despite one simple HCA near a transplant tumor after TTx, neither the EGFR nor the TGFα were upregulated in hormonal induced alterations of hepatocytes after TTx. Nevertheless, Gefitinib treatment induced a reduction of proliferative activity, suggesting that T3 induced pathways in hepatocytes, like the nuclear thyroid hormone receptor activation or related enzyme activities of i.e., cyclooxygenases, glucose-6-phosphatase or glucose-6-phosphat-dehydrogenase [16], are influenced by Gefitinib, independently of its EGFR-activity.

## 4. Experimental Section (for Details Refer to Supplementary Materials and Methods)

### 4.1. Animal Treatments

Seven hundred sixty-eight inbred male and female Lewis rats (3 months old, 150–250 g) were used in the present study. Housing of the animals was described in detail previously [14,15,16]. The animal protocols used in this work were evaluated and approved by the Committee for Animal Research (Protocol no. LALLF M-V/TSD/7221.3-1.1-023/08, 15 April 2008) and in the accordance with the German Animal Protection Law.

Animals were allocated to four different experimental settings to the chemical model of hepatocarcinogeneses after administration *N*-Nitrosomorpholine (NNM) or to three established intraportal transplantation models and to appropriate control groups, as shown in Table 4.

### 4.2. Administration of NNM

Male Lewis rats were fed NNM (Sigma Aldrich, Darmstadt, Germany) orally in a low dose (5 mg/kg body weight) daily throughout the experiment.

### 4.3. Transplantation Models

#### 4.3.1. Diabetes Induction and Intraportal Pancreatic Islet Transplantation (PTx)

Diabetes was induced by streptozotocin (80 mg/kg body weight) prior to the intraportal transplantation of 400–450 pancreatic islet grafts.

#### 4.3.2. Thyroidectomy and Intraportal Transplantation of Thyroid Follicles (TTx)

Rats were thyroidectomized before intraportal transplantation of isolated, isologous thyroid tissue pieces.

#### 4.3.3. Ovariectomy and Intraportal Transplantation of Ovarian Fragments (OTx)

Female, ovariectomized rats received isolated, isologous grafts of small fragments of ovarian tissue.

#### 4.3.4. Control Groups

Male and female Lewis rats, non-diabetic, not ovariectomized or thyroidectomized and without transplantation procedures or NNM administration were either given Gefitinib as the experimental groups or left untreated.

#### 4.3.5. Gefitinib Treatment

Gefitinib (Iressa^®^, Astra Zeneca, Wedel, Germany) was administered daily and orally for two weeks (with 20 mg/kg) or three and nine months, respectively (with a reduced dose of 10 mg/kg), to respective groups as shown in Table 1.

#### 4.3.6. Application of the Nucleoside Analog 5-Bromo-2′-deoxyuridine (BrdU)

Seven days before sacrifice, osmotic minipumps (Osmotic Pump Model 2ML1, Alzet, Alza Corp., Palo Alto, CA, USA) filled with 40 mg of BrdU (Sigma Aldrich, Heidelberg, Germany) were implanted in one third of the animals of each group.

#### 4.3.7. Animal Sacrifices and Tissue Processing

Rats were sacrificed at 3 weeks, 3 months, 6 months, 12 months and 24 months, respectively, post transplantation. After perfusion fixation, the liver was removed and cut into slices of 1–2 mm thickness. All macroscopically visible lesions (>2 mm) and additional 10 liver slices were embedded in paraffin. Slides of 2–3 μm thickness were cut and stained by H&E and the periodic acid Schiff (PAS) reaction.

#### 4.3.8. Immunohistochemistry

Formalin-fixed, paraffin-embedded or frozen tissue serial sections were stained for TGF-α, EGFR, BrdU, sodium-glucose-transporter protein 1 (SGLT1), phosphorylated mTOR (p-mTOR), phosphorylated 4 eukaryotic translation initiation factor 4E binding protein 1 (p-4EBP1), Ras, and extracellular related kinase (PanERK). 

#### 4.3.9. Morphologic Investigations 

FAH in different models were classified according to Bannasch and Zerban [43] into glycogen storing foci, basophilic foci, amphophilic foci, and amphophilic-tigroid cell foci [16]. Furthermore, frequency and amount of transplant associated HCAs, HCCs and hyperplastic transplants or transplant tumors, respectively, were assessed. BrdU labeling indices (BrdU-LI) and volume fraction determination in hepatocellular lesions and extrafocal liver tissue were determined as described earlier [14,44]. Expression levels of immunostaining were scored as product of % of hepatocytes (factors of 0 = 0%; 1 = 1%–<10%; 2 = 10%–<75%; 3 = 75%–100%) and intensity of membranous/cytoplasmatic staining result (0 = no staining; 1 = weak; 2 = medium; 3 = strong). A product difference between groups of ≥2 was defined as upregulation or downregulation, respectively. 

### 4.4. Statistical Analysis

The Wilcoxon–Mann–Whitney *U* test was applied to determine differences regarding the BrdU-LIs in intraindividual and interindividual comparisons and in cell proliferation assays. Differences regarding the number of tumor bearing animals were assessed via Fisher’s exact test. *p*-Values of less than 0.05 were considered statistically significant.

## 5. Conclusions

EGFR inhibition by Gefitinib also diminishes chemical and hormonal induced hepatocarcinogenesis at the level of initiation in the non-cirrhotic liver, as quantity and proliferative activity of different types of pre-neoplastic liver lesions are decreased. By contrast, progression to neoplastic hepatocellular tumors, even if reduced in the NNM model, is not prevented. This indicates only a partial relevance of the EGFR signaling pathway in later stages of hepatocarcinogenesis, where multiple oncogenic pathways are known to be activated, but should be considered for combined therapy with conventional cytostatic drugs or other targeted agents.

## Figures and Tables

**Table:** (**a**)

Duration of NNM Administration	Gefitinib Administration	N	BrdU-LI FAH, %	BrdU-LI Extrafocal Liver Tissue, %
3 months		7	68.26 ± 6.82 ^§^	18.07 ± 2.72
3 months	2 weeks HD	7	60.80 ± 2.80 ^§^	18.33 ± 2.88
3 months	3 months LD	7	55.49 ± 3.00 ^§^	12.53 ± 1.29

**Table:** (**b**)

Duration of NNM Administration	Gefitinib Administration	N	BrdU-LI FAH %	BrdU-LI HCA %	BrdU-LI HCC %
6 months		6	85.50 ± 1.90	89.73 ± 1.13	97.14 ± 0.32
6 months	2 weeks HD	7	73.89 ± 3.17	81.03 ± 2.70	95.49 ± 0.82
6 months	3 months LD	7	70.36 ± 1.75 ^#^	76.03 ± 1.75	92.67 ± 1.58

**Table:** (**c**)

Duration of NNM Administration	Gefitinib Administration	N	Volume Fraction FAH %	Volume Fraction HCA %	Number of HCC per Animal
3 months		14	7.25 ± 0.90	-	-
3 months	2 weeks HD	14	4.92 ± 0.87 ^#^	-	-
3 months	3 months LD	15	1.58 ± 0.28 ^#^	-	-
6 months		12	85.86 ± 0.67	10.36 ± 0.89	7.93 ± 0.92
6 months	2 weeks HD	15	84.82 ± 0.83	10.51 ± 0.56	6.53 ± 1.61
6 months	3 months LD	15	88.01 ± 0.57 ^#^	5.43 ± 0.57 ^#^	4.87 ± 0.77 ^#^

Rats were subjected to daily administration of NNM (5 mg/kg body weight). Gefitinib was administered daily and orally for two weeks (HD, high dose of 20 mg/kg) or three months and nine months, respectively (LD, low dose of 10 mg/kg). BrdU was administered for the duration of one week before sacrifice via an osmotic minipump. Data are expressed in percent (mean ± SEM). N—number of evaluated animals. BrdU-LI—BrdU labeling index; FAH—foci of altered hepatocytes; HCA—hepatocellular adenoma; HCC—hepatocellular carcinoma. ^§^ *p* < 0.05 vs. extrafocal liver tissue; ^#^ vs. untreated controls.

**Table 2:** The effect of gefitinib treatment on the proliferative activity of pre-neoplastic liver lesions and extrafocal liver tissue in hormonal hepatocarcinogenesis.

Treatment Procedure	Time after Tx	Gefitinib Administration	N	BrdU-LI FAH %	BrdU-LI Extrafocal Liver Tissue, %
PTx	3 weeks		4	10.7 ± 0.99 ^§^	3.48 ± 0.77
	3 weeks	2 weeks HD	4	5.74 ± 0.49 ^§^	1.25 ± 0.26
	6 months		5	6.06 ± 0.44 ^§^	1.17 ± 0.08
	6 months	2 weeks HD	6	2.27 ± 0.13 ^§,#^	0.60 ± 0.03 ^#^
	6 months	3 months LD	6	3.55 ± 0.22 ^§^	0.74 ± 0.04
	12 months		5	2.43 ± 1.39	0.35 ± 0.27
	12 months	2 weeks HD	9	1.26 ± 0.44 ^§^	0.09 ± 0.05
	12 months	3 months LD	7	1.55 ± 0.51	0.49 ± 0.20
	24 months		4	10.50 ± 6.94	6.69 ± 2.80
	24 months	9 months LD	3	19.20 ± 5.82	5.33 ± 3.78
OTx	3 months		7	29.79 ± 2.68 ^§^	2.66 ± 0.39
	3 months	2 weeks HD	6	12.67 ± 2.64 ^§,#^	2.67 ± 1.59
	12 months		6	16.52 ± 2.47 ^§^	5.68 ± 0.10
	12 months	2 weeks HD	7	8.73 ± 0.88 ^§,#^	5.12 ± 0.94
	12 months	3 months LD	4	13.55 ± 2.50	6.29 ± 0.49
	24 months		6	6.50 ± 1.56	2.26 ± 0.58
	24 months	9 months LD	5	11.10 ± 1.77	6.35 ± 5.22
TTx	3 months		7	12.25 ± 1.33 ^§^	2.84 ± 0.53
	3 months	2 weeks HD	8	7.69 ± 1.07 ^#^	6.61 ± 1.63 ^#^
	12 months		8	11.04 ± 1.45 ^§^	1.07 ± 0.18
	12 months	2 weeks HD	6	11.36 ± 1.55 ^§^	0.69 ± 0.28
	12 months	3 months LD	6	10.62 ± 1.91 ^§^	2.31 ± 1.08
	24 months		7	9.33 ± 2.67 ^§^	1.58 ± 0.90
	24 months	9 months LD	9	6.34 ± 1.23 ^§^	1.13 ± 0.33

Proliferative activity foci of altered hepatocytes (FAH) and extrafocal tissue, determined as BrdU labeling index (BrdU-LI) after intraportal transplantation of pancreatic islets (PTx), ovarian fragments (OTx) or thyroid follicles (TTx) at different time points and with or without administration of gefitinib (HD, high dose of 20 mg/kg body weight, LD, low dose of 10 mg/kg body weight). N—number of evaluated animals. BrdU was administered via an osmotic pump for a duration of one week before sacrifice. *p* < 0.05 ^§^ vs. extrafocal liver tissue, ^#^ vs. untreated controls.

**Table 3:** The effect of gefitinib treatment on the frequency of hepatocellular tumors in hormonal hepatocarcinogenesis.

Treatment Procedure	Time after Tx	Gefitinib Administration	Rats with HCA	Rats with HCC	Rats with Transplant Hyperplasia or Tumors
PTx	12 months		16 (34)	0 (34)	0
	12 months	2 weeks HD	8 (17)	0 (17)	0
	12 months	3 months LD	11 (17)	0 (17)	0
	24 months		5 (19)	1 (19)	0
	24 months	9 months LD	3 (10)	1 (10)	0
OTx	12 months		0 (15)	0 (15)	3 (15)
	12 months	2 weeks HD	1 (16)	0 (16)	6 (16)
	12 months	3 months LD	1 (16)	0 (16)	7 (16)
	24 months		9 (20)	1 (20)	10 (20)
	24 months	9 months LD	9 (21)	1 (21)	12 (21)
TTx	12 months		1 (15)	0 (15)	0 (15)
	12 months	2 weeks HD	2 (14)	0 (14)	1 (14)
	12 months	3 months LD	2 (13)	0 (13)	1 (13)
	24 months		3 (15)	0 (15)	2 (15)
	24 months	9 months LD	8 (15)	0 (15)	2 (15)

Frequency of transplant associated hepatocellular adenomas (HCA), carcinomas (HCC) and transplant hyperplasia or tumors after intraportal transplantation of pancreatic islets (PTx), ovarian fragments (OTx) or thyroid follicles (TTx) at different time points and with or without administration of gefitinib (HD, high dose of 20 mg/kg body weight, LD, low dose of 10 mg/kg body weight). The number of investigated animals is given in parentheses.

**Table 4:** Experimental design of investigated models of hepatocarcinogenesis.

Treatment Procedure	Sacrifice Time Point	Gefitinib Administration	N		Sacrifice Time Point	Gefitinib Administration	N
Experimental groups				Control groups			
PTx (male)	3 weeks	-	8	male	3 weeks	-	10
	3 weeks	2 weeks HD	12		3 weeks	2 weeks HD	10
	6 months	-	10		3 months	-	15
	6 months	2 weeks HD	14		3 months	2 weeks HD	15
	6 months	3 months LD	12		3 months	3 months LD	15
	12 months	-	34		6 months	-	26
	12 months	2 weeks HD	17		6 months	2 weeks HD	25
	12 months	3 months LD	17		6 months	3 months LD	25
	24 months	-	19		12 months	-	11
	24 months	9 months LD	10		12 months	2 weeks HD	10
					12 months	3 months LD	10
TTx (male)	3 months	-	15		24 months	-	19
	3 months	2 weeks HD	15		24 months	9 months LD	24
	12 months	-	15				
	12 months	2 weeks HD	14	female	3 months	-	10
	12 months	3 months LD	13		3 months	2 weeks HD	10
	24 months	-	15		12 months	-	14
	24 months	9 months LD	15		12 months	2 weeks HD	9
					12 months	3 months LD	9
OTx (female)	3 months	-	15		24 months	-	21
	3 months	2 weeks HD	15		24 months	9 months LD	17
	12 months	-	15				
	12 months	2 weeks HD	16				
	12 months	3 months LD	16				
	24 months	-	20				
	24 months	9 months LD	21				
NNM	3 months	-	15				
	3 months	2 weeks HD	15				
	3 months	3 months LD	15				
	6 months	-	15				
	6 months	2 weeks HD	15				
	6 months	3 months LD	15				

Overview of all investigated experimental and control groups in short (3 weeks, and 3 and 6 months) and long term investigations (12 and 24 months) after intraportal transplantation of pancreatic islets (PTx), ovarian fragments (OTx) or thyroid follicles (TTx) or administration of *N*-Nitrosomorpholine (NNM) at different sacrifice time points (time after transplantation or beginning of NNM administration) and with or without administration of gefitinib (HD, high doses of 20 mg/kg body weight, LD, low doses of 10 mg/kg body weight) as indicated. N number of animals in respective groups.

## Supplementary Materials

Supplementary materials can be found at www.mdpi.com/1422-0067/17/10/1618/s1. Reference [45] is cited in the Supplementary materials.
