# Supplementary material for: The Epidermal Growth Factor Receptor (EGFR) Inhibitor Gefitinib Reduces but Does Not Prevent Tumorigenesis in Chemical and Hormonal Induced Hepatocarcinogenesis Rat Models
Source: Int J Mol Sci. 2016 Sep 23;17(10):1618. doi: 10.3390/ijms17101618 (PMC5085651; doi:10.3390/ijms17101618)
Supplement: Supplementary file 1 [file ijms-17-01618-s001.pdf]

Supplementary Materials: The Epidermal Growth Factor Receptor (EGFR) Inhibitor Gefitinib Reduces but Does Not Prevent Tumorigenesis in Chemical and Hormonal Induced Hepatocarcinogenesis Rat Models

Silvia Ribback, Verena Sailer, Enrico Böhning, Julia Günther, Jaqueline Merz, Frauke Steinmüller, Kirsten Utpatel, Antonio Cigliano, Kristin Peters, Maria G. Pilo, Matthias Evert, Diego F. Calvisi and Frank Dombrowski

Supplementary Materials and Methods

1. Animal Treatments

Seven hundred sixty-eight inbred male and female Lewis rats (3 months old, 150–250 g) were used in the present study. Housing of the animals was described in detail previously [14–16] and was in accordance with the guidelines of the Society for Laboratory Animal Service and the German Animal Protection Law.

Animals were allocated to four different experiments to the chemical model of hepatocarcinogenesis after administration *N*-Nitrosomorpholine (NNM) or to three established intraportal transplantation models and to appropriate control groups, as shown in Table 1.

Administration of NNM

Male Lewis rats were fed NNM (Sigma Aldrich, Darmstadt, Germany) orally at a low dose (5 mg/kg body weight) daily throughout the experiment.

2. Transplantation Models.

2.1. Diabetes Induction and Intraportal Pancreatic Islet Transplantation (PTx)

Diabetes was induced in male Lewis rats by giving a single subcutaneous dose of streptozotocin (80 mg/kg body weight) one week prior to the transplantation. Animals were considered diabetic with a blood glucose level of at least 16.7 mmol/L. Details of the transplantation procedure have been described previously [14]. Animals received 400–450 isolated, isologous pancreatic islet grafts under anesthesia (Ketamine/Xylacine 100 mg/40 mg per kg body weight) from non-diabetic donor rats, so that mild hyperglycemia persisted.

2.2. Thyroidectomy and Intraportal Transplantation of Thyroid Follicles (TTx)

Male Lewis rats were thyroidectomized two weeks before transplantation. Isolated, isologous thyroid tissue pieces from donor rats were transplanted via the portal vein. Donor animals received an iodine-poor diet two weeks before transplantation to enlarge the grafts, as described previously [16]. Anesthesia was achieved with diethyl-ether (Roth Karlsruhe, Germany).

2.3. Ovariectomy and Intraportal Transplantation of Ovarian Fragments (OTx)

Female Lewis rats received isolated, isologous grafts of small fragments of ovarian tissue from donor animals into the portal vein as described before [16,17].

In all three models, grafts were transplanted to the right part of the liver, secured by previous clamping of the left branch of the portal vein, to ensure the left part of the liver as internal control tissue.

2.4. Control Groups

Male and female Lewis rats, non-diabetic, not ovariectomized or thyroidectomized and without transplantation procedures or NNM administration were either given Gefitinib as the experimental groups or left untreated.

2.5. Gefitinib Treatment

Gefitinib was dissolved in 0.5% carboxymethylcellulose (Sigma-Aldrich, Darmstadt, Germany) administered daily and orally for two weeks (with 20 mg/kg) or three and nine months, respectively (with a reduced dose of 10 mg/kg), to respective groups as shown in Table 1.

2.6. Application of the Nucleoside Analog 5-Bromo-2′-deoxyuridine (BrdU)

Seven days before sacrifice, approximately one third of the animals of each group was anaesthetized and osmotic minipumps (Osmotic Pump Model 2ML1, Alzet, Alza Corp., Palo Alto, CA, USA) filled with 40 mg of BrdU (Sigma Aldrich, Heidelberg, Germany) were surgically implanted subcutaneously between the scapulae. These pumps continuously delivered BrdU until the animals were sacrificed.

2.7. Animal Sacrifices

Animals were killed under anesthesia as described previously [14–16]. Rats were sacrificed at the following time points: 3 weeks, 3 months, 6 months, 12 months, and 24 months. Animals that died spontaneously were incorporated in the nearest time group. These animals were not analyzed regarding proliferation activity of preneoplastic foci because of loss of tissue quality, but evaluation of the development of hepatocellular neoplasm was still possible.

2.8. Tissue Processing

Details are described previously [14]. Briefly, after perfusion fixation, the liver was removed and cut into slices of 1–2 mm thickness. All macroscopically visible lesions (>2 mm) and additional 10 liver slices were embedded in paraffin. Slides of 2–3 μm thickness were cut and stained by H&E and the periodic acid Schiff (PAS) reaction.

2.9. Immunohistochemistry

Formalin-fixed, paraffin-embedded serial sections of 1–2 μm thickness were stained for TGF-α (mouse monoclonal antibody, dilution 1:100, Merck, Darmstadt, Germany) and EGFR (mouse monoclonal antibody, dilution 1:50, Leica Biosystems, Wetzlar, Germany) in an automated immunostainer (Leica Biosystems, Wetzlar, Germany) using a DAB (diaminobenzidine) kit. Immunostain for BrdU (monoclonal antibody, dilution 1:100 overnight, DAKO, Hamburg, Germany) was performed as described earlier [14,44].

Frozen tissue was cut to 10 μm thick slices in a cryostat and stained with H & E and PAS. Serial cryostat sections were incubated with primary antibodies to sodium-glucose-transporter protein 1 (SGLT1; rabbit polyclonal, dilution 1:100 overnight; Abcam, Cambridge, UK), EGFR, TGF-α (as mentioned above), phosphorylated mTOR (rabbit monoclonal, dilution 1:50, Cell Signaling Technology Inc., Danvers, MA, USA), phosphorylated 4 eukaryotic translation initiation factor 4E binding protein 1 (p-4EBP1; rabbit monoclonal, dilution 1:25, Cell Signaling technology Inc., Danvers, MA, USA), Ras (mouse monoclonal, dilution 1:50, BD Biosciences, San Diego, CA, USA), extracellular related kinase (PanERK, mouse monoclonal, dilution 1:50, BD Biosciences, San Diego, CA, USA). Endogeneous peroxidase was quenched with 1% hydrogen peroxide, and positive reactivity of primary antibodies was identified using the Ultravision LP detection system AP polymer and Fast Red chromogen or HRP polymer and DAB as chromogen substrate (Thermoscientific, Waltham, MA, USA). Immunohistochemical signal intensity in FAH was

estimated semi-quantitatively comparing FAH with corresponding surrounding unaltered liver tissue. Negative controls were stained without a primary antibody.

2.10. Morphologic Investigations

FAH in different models were classified according to Bannasch and Zerban [42] into glycogen storing foci, basophilic foci, amphophilic foci, and amphophilic-tigroid cell foci [16]. Furthermore, frequency and amount of transplant associated hepatocellular adenomas (HCAs) and hepatocellular carcinomas (HCCs) were assessed. HCA were diagnosed when the lesions were sharply limited and compressed the surrounding liver parenchyma. HCCs were defined if the lesion exhibited trabeculae thicker than three cell layers in at least two separate areas, revealed high numbers of mitotic figures, and had a diameter larger than 5 mm. Sporadic HCA that were not associated to transplants were not included in the assessment; sporadic HCC did not occur in the study. Hyperplastic transplants were defined in case of OTx as stromal overgrowth without follicles and granulosa cell layer, without cellular atypia [15]. Transplant tumors in the case of TTx were defined as neoplastic proliferations of thyreocyts with loss of colloid and organoid follicle structure, nuclear atypia, high mitotic activity and a diameter of at least 5 mm. BrdU labeling indices (BrdU-LI) in hepatocellular altered lesions and extrafocal liver tissue were determined as described earlier [43,44]. Volume fraction determination of FAH in NNM-induced hepatocellular lesions was estimated from their area fraction in H&E staining at 100× magnification, determined by the point-counting stereological technique with a monitor grid as described by Weibel [45] using a NIKON DS-2MV digital camera and the NIKON NIS Elements Imaging Software Package 4.0. At least 11,050 points were counted within 50 visual fields per section, representing an area of 37.5 mm^2. According to Weibel, these area fractions are equivalent to volume fractions. Furthermore, frequency and quantity of HCA and HCC per animal was estimated.

Expression levels of immunostaining were scored as product of percent of hepatocytes (factors of 0 = 0%; 1 = 1%–<10%; 2 = 10%–<75%; 3 = 75%–100%) and intensity of membranous/cytoplasmatic staining result (0 = no staining; 1 = weak; 2 = medium; 3 = strong). A product difference between groups of ≥2 was defined as upregulation or downregulation, respectively.

2.11. Statistical Analysis

The Wilcoxon–Mann–Whitney *U* test was applied to determine differences regarding the BrdU-LIs in intraindividual and interindividual comparisons and in cell proliferation assays. Differences regarding the number of tumor bearing animals were assessed via Fisher's exact test. *p*-Values of less than 0.05 were considered statistically significant.
